# Mental health capacity building in Mali by training rural general practitioners and raising community awareness

**DOI:** 10.11604/pamj.2021.38.389.26838

**Published:** 2021-04-21

**Authors:** Oumar Poudiougou, Pierre-Emile Bruand, Pakuy Pierre Mounkoro, Jean-Michel Gaglione, Karamoko Nimaga, Mansour Sy, Clotilde Vincent, François Calas, Amy Fall-Ndao, Lucie Petiteau, Nicole Hanssen, Djamirou Dossa, Farid Boumédiène, Pierre-Marie Preux, Arouna Togora

**Affiliations:** 1 Santé Sud, Bamako, Mali,; 2 Global Health, Sanofi, Gentilly, France,; 3 Department of Psychiatry, Point G Hospital, Bamako, Mali,; 4 Department of Psychiatry, Martigues Hospital, Martigues, France,; 5 Association of Rural Physicians in Mali, Markacoungo, Mali,; 6 Santé Sud, Marseille, France,; 7 Global Health, Sanofi, Dakar, Senegal,; 8 Institut National de la Santé et de La Recherche Médicale (INSERM), Université Limoges, Centre Hospitalier Universitaire Limoges, Institut de Recherche pour le Développement (IRD), U1094 Tropical Neuroepidemiology, Institute of Epidemiology and Tropical Neurology, Institut Génomique-Environnement-Immunité-Santé et Thérapeutiques (GEIST), Limoges, France

**Keywords:** Capacity building, general practitioners, mental health, public awareness, training

## Abstract

**Introduction:**

despite the high prevalence and significant burden of mental disorders, they remain grossly under-diagnosed and undertreated. In low-income countries, such as Mali, integrating mental health services into primary care is the most viable way of closing the treatment gap. This program aimed to provide a mental health training intervention to rural general practitioners (GPs), to organize community awareness activities, and to evaluate the impact on mental health knowledge and through the number of new patients diagnosed with mental disorders and managed by these general practitioners.

**Methods:**

a pre-test/post-test design and the monthly monitoring of the number of new patients diagnosed with mental disorders by the trained GPs were used to evaluate the effect of the training interventions (two face-to-face group training workshops followed by individual follow-up supervisions) and of the community awareness activities.

**Results:**

the mean knowledge score of the 19 GPs who completed the initial 12-day group training raised from 24.6/100 at baseline, to 61.5/100 after training (p<0.001), a 150% increase. Among them, sixteen completed the second 6-day group training with a mean score increasing from 50.2/100 to 70.1/100 (p<0.001), a 39.6% improvement. Between July 2018 and June 2020, 2,396 new patients were diagnosed with a mental disorder by the 19 GPs who took part in the program.

**Conclusion:**

despite limited data regarding the effect of the community awareness component at this stage, the findings from this study suggest that the training intervention improved GPs' knowledge and skills, resulting in a significant number of new patients being identified and managed.

## Introduction

The worldwide prevalence of mental disorders is high: in a systematic review and meta-analysis of 174 studies (conducted in 26 high-income countries and 37 low-and middle-income countries (LMICs)) [1], the 12-month prevalence for adults was 17.6% (95% confidence interval: 16.3-18.9%) and the lifetime prevalence 29.2% (25.9-32.6%). In sub-Saharan Africa (8 studies, 6 countries), the 12-month prevalence was 10.8% (7.2%-15.9%). In Mali, the only published data [2] are for depressive disorders (3.6%) and anxiety disorders (2.6%) (crude prevalence rates, not age-standardized) and these would suggest that the prevalence of mental disorders is just as high as in other countries. Despite a high prevalence, mental disorders remain under-diagnosed and under-treated especially in LMICs: the World Health Organization (WHO) estimates that 76-85% of people with severe mental disorders living in LMICs receive no treatment [3]. In Mali, as in most LMICs, mental health is under-resourced with very low specialized workforce: for instance, there are only 0.03 psychiatrists per 100,000 population [4] compared to 10.54 in the US [5] or 20.91 in France [6]. Moreover, these psychiatrists are based in Bamako, the capital, whilst approximately 60% of the population live in rural areas [7]. Although the WHO has called for mental health services to be integrated into primary care [8,9], mental health remains in most countries, and especially in LMICs, one of the most neglected topics in the training curriculum of frontline health workers with very little time dedicated to it [10,11]. For instance, in Myanmar, only one percent of the training for medical doctors is devoted to mental health [10].

As a result, GPs leave medical school with insufficient knowledge and negative attitudes towards mental disorders: a study conducted in India [12] showed that only 28% of GPs were able to name three common symptoms of psychosis, while 59% believed that “mental health problems were a sign of personal weakness”. Another barrier to mental health care in LMICs is the low mental health literacy in the general population: misunderstanding of the symptoms is frequent, beliefs and prejudices regarding mental disorders common. These lead to reluctance to seek care from healthcare providers, stigmatization, discrimination and exclusion of the patients and their families [13-16]. In Mali, if mental health care is to be effectively integrated into primary care, rural GPs, who are pivotal primary care providers, need to acquire the relevant knowledge and skills to be able, in their own communities to identify, diagnose, manage and support people experiencing mental disorders, thus contributing to the humanization of the management of these diseases. The program we implemented aimed to provide a mental health training intervention to a group of rural GPs in Mali to diagnose mental health issues and treat them at primary health care level, and to evaluate the effect of this training.

## Methods

**Study design:** this program involved a pre-test/post-test design to evaluate the effect of two face-to-face group mental health training workshops combined with individual follow-up supervision sessions, as well as the implementation of mental awareness activities directed towards the general population, and the monthly monitoring of the number of new patients with mental disorders diagnosed and managed by the trained GPs. Approval was received by the Direction Nationale de la Santé, Ministry of Health, Republic of Mali in August 2017 to conduct these activities.

**Intervention:** this 2-year intervention program which was implemented between June 2018 and June 2020, was based on three main groups of activities: capacity building, public awareness and advocacy, monitoring and evaluation.

**Capacity building:** the aim was to leverage an existing network of rural GPs, by training 18 of them to diagnose and manage mental disorders, and to also train 8 of them so that they could, in turn, train other GPs. The initial face-to-face training was a 12-day session held in Bamako from 25 June until 7 July 2018. This initial training combined didactic presentations, questions and answers (Q and A) sessions, group workshops facilitated by experienced psychiatrists from the Point G University Hospital and from Martigues Hospital, France, as well as clinical hands- on- experience with two days spent at the Psychiatric Department of the Point G Hospital. The second training was a 6-day session which was held a year later (2-7 July 2019) also in Bamako. It included a practical part with four mornings of consulting sessions, with the support of the psychiatrists from the Psychiatric Department of the Point G Hospital and from Martigues Hospital, and a theoretical part in the afternoon with didactic presentations, Q and A session and sharing of the experience from the morning consultations. After each of these face-to face group trainings, individual follow-up supervision and mentoring sessions (one and a half days for each of the trainees) were also organized at each GP health centre, during which, consultations were supervised, and medical records were discussed. These took place through February-March 2019, November-December 2019, and June 2020. In October 2019, eight of the trained GPs were also trained to become trainer of GPs and other health workers.

**Public awareness and advocacy:** a workshop was organized in September-October 2018 with the participation of the National Center of Information Education and Communication for Health (CNIECS - Centre National d´Information, d´Education et de Communication pour la Santé), psychiatrists from the Point G hospital, a representative of the Malian federation of traditional healers and herbalists (FEMATH), a representative of the national federation of community health associations (FENASCOM), a representative of the health communicators network and 3 GPs from the rural network. It led to the development of several educational tools: an educational flipchart with 11 key messages to be used by GPs and community health workers (CHWs) to inform, raise awareness and educate the local population as well as radio messages in 3 of the local languages (Bambara, Peulh and Soninké). Between February 2019 and March 2020, there were radio broadcasts 4 times a week through 13 community radio stations, and 3 times a week through 2 regional stations. From May 2019, CHWs were trained to raise awareness, and advocacy meetings have been organized with traditional healers as well as religious and local leaders.

**Monitoring and evaluation:** as the number of patients with mental disorders seen by the trained GPs was an important indicator to monitor, collecting monthly data was included as part of the activities to perform. From July 2018, the project manager has been following-up to ensure that every GP would send each month via whatsApp their report including the number of total new consultations, the number of new patients diagnosed with mental disorders as well as the number of these patients followed-up.

**Output and outcome measures:** the change in knowledge has been measured through pre-and post-training questionnaires. For the 2018 training, the questionnaire included 40 questions with 0.5 point per question to get a total maximum score of 20, whereas in 2019, there were 20 questions with 1 point per question to get a total maximum score of 20. For the current analysis, these have been converted into a global score out of a maximum of 100. Beyond a global score, sub-scores were also calculated around specific disease areas and post-training scores improvement were expressed as percentage increases compared to pre-training scores. Other outcomes, such as the number of patients diagnosed with mental disorders and managed by the newly trained GPs, have been reported by each GP every month. They have been split into different diseases: psychoses, mood disorders, anxiety disorders, post-traumatic stress disorder (PTSD), addictions, autism and dementia. Other outputs such as the number of CHWs trained, number of people attending awareness meetings, number of traditional healers, religious and other local leaders met have also been reported on a monthly basis by GPs.

**Study area:** this intervention program has been implemented across 19 GP community practices and rural clinics (with an estimated total practice population base of 216,000 people) in rural areas in six regions of Mali: Kayes, Koulikoro, Ségou, Sikasso, Mopti and Timbuktu.

**Statistical analysis:** descriptive analysis has been performed for proportions, percentages, means and their corresponding standard deviations. Knowledge has been expressed as a score out of a maximum of 100 and the Wilcoxon-Mann-Whitney test for paired samples has been used to specifically examine changes between pre- and post-training. For all statistical analyses, p value of less than 0.05 has been set to determine statistical significance.

## Results

**Figure 1:**
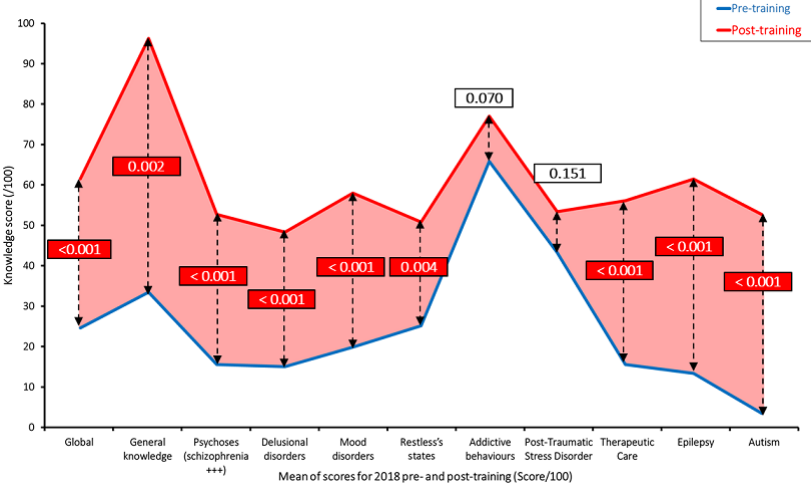
2018 training - pre and post mean scores (/100) - global and for individual areas-n =19 GPs - p values from Wilcoxon-Mann-Whitney test

**Figure 2:**
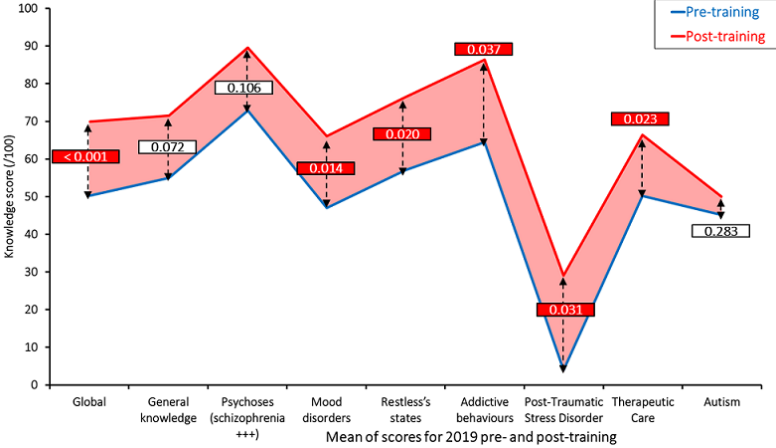
2019 training - pre and post mean scores (/100) - global and for individual areas-n =16 GPs - p values from Wilcoxon-Mann-Whitney test

**Training and knowledge:** nineteen GPs completed the initial face-to-face group training in June-July 2018. Their mean score increased from 24.6/100 before training, to 61.5/100 after training (p<0.001), i.e. a 150% increase compared to the pre-training score. As shown in Figure 1, the increase in knowledge was significant in all areas apart from addictive behaviors and PTSD, where baseline scores seemed actually higher than for other areas. Individual scores increased for each of the 19 participants: scores ranging from 11/100 to 47/100 at baseline raised to 41/100 to 84/100 post-training, a 53% to 365% increase. On site individual follow-up training and supervision took place in February-March 2019 with 70 supervised consultations (1 to 30 per health center) and 340 patients medical records reviewed (8 to 146 per health center). Following the death of one of the GPs from the initial group of 19 GPs in March 2019, and the fact that two other GPs left the program between December 2018 and February 2019 to start a psychiatry degree (Diplôme d´Etude Spécialisée - DES) at the University of Bamako, 16 GPs completed the second face-to-face group training in July 2019. Their mean score went from 50.2/100 before this second training to 70.1/100 after the training (p<0.001), a 39.6% improvement compared to the second session pre-training score. As shown in Figure 2, the increase in knowledge was significant in all areas apart from the general knowledge, psychoses and autism questions. Individual scores increased for all 16 GPs who did the second training: pre-training scores ranged from 39.25/100 to 71/100, while post-training scores were between 47.5/100 and 86/100. The percentage increases for the individual scores were between +6% and +119%. Thirteen GPs benefited from a second round of on-site individual follow-up training, which included 99 supervised patient consultations. A 20-item questionnaire (covering areas such as: attitudes towards the patient and their family, interviewing technique and documenting, examining, advising the patient) was actually used by the psychiatrist supervising the consultation to evaluate the skills of the GPs while they were seeing the patient and their family. The scores ranged between 80% and 95% (results not shown), with two areas of improvement where most GPs didn´t seem to have reached the required level of competency: psychological assessment, as well as emphasizing the importance of psychosocial support by the family and the community. Due to the security situation in Mopti and Timbuktu, a group follow-up session had to be organized for the three remaining GPs during which, 11 of their patient cases were reviewed with the psychiatrist and facilitator.

**Awareness and advocacy:** between May 2019 and June 2020, 183 CHWs in the six targeted areas were equipped with educational flip-charts and trained to inform, raise awareness, educate communities regarding mental health and to address the many prejudices and the exclusion which people with mental disorders are often suffering from. Overall, approximately 76,000 people have been reached by awareness activities led by CHWs or GPs. In total, there were 336 community and regional radio broadcasts between February 2019 and June 2020, reaching an estimated 314,740 people in the six regions. Overall, GPs have met with fifty traditional healers and local leaders, mainly in Timbuktu (n=9) and in Mopti (n=10), to raise awareness about mental disorders and advocate for a better understanding of the diseases, their symptoms, and for greater collaboration with traditional healers. There have been some positive collaborative experiences, in particular regarding patients with mental disorders who have been referred by traditional healers to GPs in Gakoura, in Séguela (Kayes Region), in Markacoungo (Koulikoro Region), and in Koumantou (Sikasso Region). The most extensive collaboration has taken place in Markacoungo, particularly in Siratiguila, where the team came across a traditional healing centre, housing approximately 100 patients with various mental disorders, most of whom were being kept in inhumane conditions, chained up in a shed. To improve and humanize the care of these patients, an initiative involving a medical student and the traditional healer is being implemented and will be the topic of a thesis in medicine.

**Figure 3:**
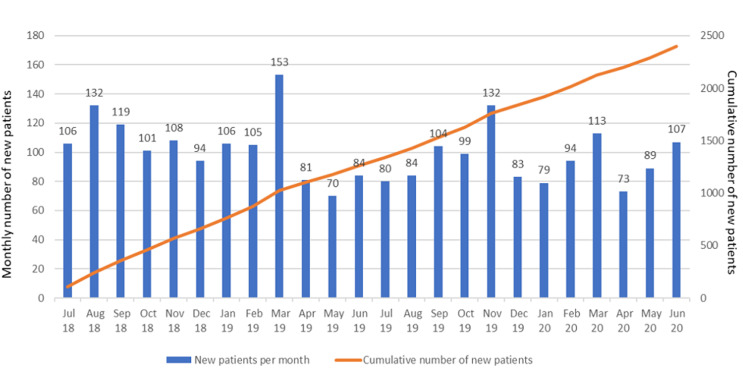
monthly and cumulative number of new patients diagnosed by the trained GPs between July 2018 and June 2020

**Figure 4:**
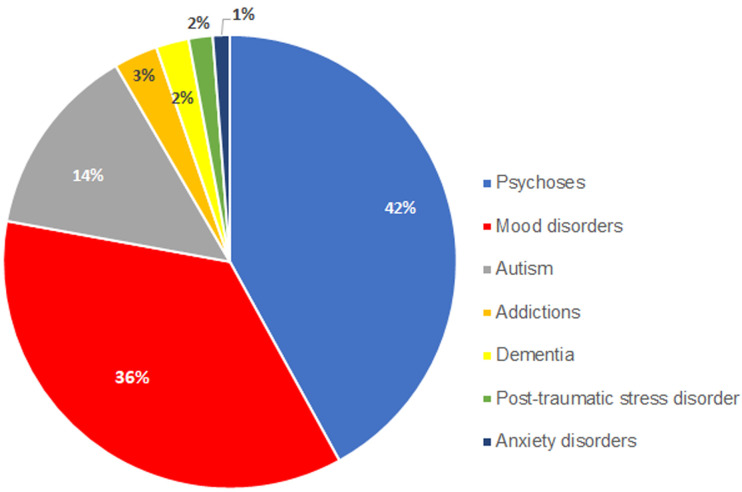
diseases diagnosed among the 2,396 new patients (%)

**New patients diagnosed:** between July 2018 and June 2020, out of the 228,238 patient visits recorded by the 19 GPs who took part in the program, there were 2,396 new patients diagnosed with a mental disorder cumulatively (Figure 3). In June 2020, there were 2,293 who were still followed-up. Among the patients who were newly diagnosed, a wide range of mental disorders have been identified: psychoses (42.0%), mood disorders (35.9%) and autism (13.7%) being the most frequent ones (Figure 4). The number of patients newly diagnosed each month remained relatively stable over the two-year period ranging from 70 to 132, although from February 2019, there were only 16 GPs still involved in the program.

## Discussion

Studies have shown the high percentage of patients presenting to primary healthcare centers with mental disorders. A study conducted in 2004 in an isolated rural district of Sobral, Brazil, found that approximately 60% of primary care patients had mental disorders or significant mental distress, and around 40% had medically unexplained symptoms [17]. In another study of 1,146 patients with major depression recruited across 15 primary care centers in 14 countries on 5 continents, 69% of them reported only physical symptoms as the reason for their physician visit [18]. Therefore, GPs have a pivotal role to play in diagnosing and managing these patients. With the worldwide spread of the COVID-19 pandemic, its associated restrictions and containment measures, such as lockdowns, physical distancing, isolation and its resulting impact on the economy, with unemployment soaring and social inequalities being exacerbated, it is anticipated that the incidence of mental disorders will increase, and that among people with pre-existing mental disorders, their mental health status will worsen [19]. Frontline healthcare workers will therefore be even more frequently facing patients with mental disorders in their daily practice. As stated by the WHO “Integrating mental health services into primary care is the most viable way of closing the treatment gap and ensuring that people get the mental health care they need” [8]. Hence the recommendation of adequate training of primary care workers in diagnosing and treating people with mental disorders as highlighted in the WHO Mental health action plan (2013-2020) [3] and the WHO mental health gap Action programme (mhGAP) [9]. The results of this work suggest that the training implemented in Mali led to an improvement in the mental health knowledge of the GPs who were trained, with significant and substantial increases in mean scores achieved during both the first and second training sessions (respectively 150%, p<0.001 and 39.6%, p<0.001), and a relatively high mean score reached at the end of the second training session (70.1/100). In addition, for both training sessions, individual knowledges scores for all GPs increased.

It is also worth noting that the baseline knowledge score, prior to GPs starting the initial training, was low (24.6/100 on average, with individual baseline scores ranging between 11/100 and 47/100) confirming the inadequate training of GPs in mental health and the knowledge gap, which have been highlighted in other countries [10,11]. We cannot, however, make any comparison between the score immediately after the initial training (61.5/100) and the score prior to the second training (50.2/100), as the questionnaires which were used were different, the content of the second training being quite different from the first one. Beyond the increase in knowledge score, the high number of new patients with mental disorders (n=2,396) which were diagnosed and managed by these trained GPs over the 2-year program would suggest that new competencies have been put into practice. This has also been confirmed by the various rounds of on-site individual follow-up training and supervision; the psychiatrist, GP facilitator and project manager who conducted these, noticed, in particular during the second round, “a notable improvement in the GPs skills in the management of a number of mental disorders”, as mentioned in their reports, and as also shown in the questionnaires completed for each consultation evaluated. The fact that two of the GPs who went through the 1^st^ training intervention decided to complete a psychiatry degree at the Medicine and Odonto-Stomatology Faculty (FMOS) of the University of Sciences, techniques and Technologies of Bamako (USTTB) is a sign of the interest generated and of the relevance of the mental health training for GPs. It is also worth emphasizing that, over the two-year intervention period, the number of patients newly diagnosed each month remained relatively stable (mean of 100 patients per month ± 20 SD) (Figure 3), despite the fact that 3 GPs (the two mentioned above and the one deceased) left the program only 5 to 7 months after its start. This might suggest that the confidence of the trained GPs has been constantly reinforced throughout the various training interventions, and their willingness to apply the skills learned has not diminished over time.

The high percentage of psychoses diagnosed (42.0%) Figure 4, might reflect the devastating impact that this group of diseases can have on the lives of patients and their families and even of communities. There are several limitations to this study, including the lack of a control group. Although there are no obvious reasons for knowledge to change over time without any form of learning, without a control group one cannot confidently attribute the change in knowledge, or the number of new patients diagnosed and managed by the trained GPs, solely to the training. When it comes to mental disorders, beyond the knowledge gap, prejudices and negative attitudes have been shown to be a key issue among primary health workers [12,13,20]. It would have been useful to also evaluate potential changes in beliefs and attitudes among the trained GPs by including relevant questions in the pre- and post- training questionnaires. Another limitation is that, for the various awareness activities, there have not been any outcome measures, even if data were collected regarding outputs (such as the number of broadcasts of the radio messages, the number of people reached through the activities). It would have been very useful to evaluate spontaneous and prompted recalls of key messages by the population after attending awareness meetings held by CHWs or listening to radio broadcasts, as well as their understanding and agreement with these messages. Similarly capturing the number of new patients presenting to GP practices as a result of attending a meeting held by a CHW or listening to one of the radio stations broadcasting the mental health messages, would have been helpful to assess the effectiveness of these activities. Although GPs have reported that they have seen an increase of patients seeking help after the start of these awareness activities, there is a lack of objective data to confirm it. The number of patients diagnosed with mental disorders has been self-reported by each GP, with no random audit to ensure that these reports were aligned with those in the medical records of the GP practice, which is also another limitation of this project.

## Conclusion

Despite some gaps in the evaluation of this program, it demonstrated positive effects on the knowledge and skills of GPs who were trained, and on the self-confidence of those GPs to diagnose and manage patients with mental disorders, as reflected by the number of new patients with mental disorders diagnosed and managed each month. Strategies to expand this program are currently being investigated, including a scaling-up leveraging the 8 GPs who have been trained to become trainers, combined with eLearning modules.

**Funding:** financial support for this work was provided by Sanofi Global Health Programs.

### What is known about this topic

- Mental disorders remain largely under-diagnosed and under-treated in Low and Middle-income Countries (LMICs), and in particular in sub-Saharan Africa, resulting in a very significant burden;
- In Mali, as in most LMICs, mental health is under-resourced, with very low specialized workforce, concentrated in Bamako, the capital city;
- In addition, primary healthcare professionals receive insufficient training to diagnose and manage people with mental disorders, making it difficult to integrate mental health services into primary care, as recommended by the World Health Organization.

### What this study adds

- Our study suggests that a short mental health training intervention for rural general practitioners can improve GPs´ knowledge and skills, and result in a significant number of new patients being diagnosed and managed;
- Over a two-year period, the two face-to-face group training sessions on mental health, combined with individual follow-up supervision and mentoring sessions resulted in improved knowledge scores and improved skills. The effect on the number of patients newly diagnosed each month by the trained GPs remained sustained over the two-year intervention period.
